# Shortened modified look-locker inversion recovery for myocardial T1 mapping in healthy volunteers: determination of reference T1 relaxation times at 3 Tesla MR according to different contrast injection methods

**DOI:** 10.1186/1532-429X-15-S1-P122

**Published:** 2013-01-30

**Authors:** Yon Mi Sung, Hwanseok Yong

**Affiliations:** 1 Department of Radiology, Gachon University Gil Hospital, Incheon, Republic of Korea; 2 Department of Radiology, Korea University Guro Hospital, Seoul, Republic of Korea

## Background

Reference pre- and post-contrast T1 values of myocardium have been reported at 1.5T but those values at 3T have not been fully established. Additionally, the physical properties of gadolinium contrast agents significantly affect the myocardial voxel T1 value. There have been two different contrast injection methods in the literatures. The aim of our study was to establish pre- and post-contrast reference T1 relaxation times of normal myocardium at 3 Tesla MR using shortened modified look-locker inversion recovery (sh-MOLLI) and to assess the effects of contrast injection methods on T1 values.

## Methods

Myocardial T1 mapping at 3 Tesla MR was obtained using sh-MOLLI from 22 healthy volunteers (15 males and 7 females, mean age, 30.9 ± 4 years), who were randomly assigned to group 1 (n = 11) and group 2 equally. The dose of contrast agent was divided to allow perfusion protocols with 60 sec delay before the second injection in group 2, whereas, the single injection of same dose in group 1. For each patient, T1 mappings at three short-axis levels were performed consecutively at pre-contrast, 2 min, 5 min, 10 min, 15 min, 20 min, 25 min and 30 min after the single injection in group 1 and the second injection in group 2. T1 values of myocardium were measured in six segments per each level and slice-averaged myocardial T1 relaxation times were calculated.

## Results

Group 1 had shorter contrast-enhanced myocardial T1 relaxation times compared to group 2 without statistical significance (p = 0.255). Mean pre- and post-contrast slice-averaged myocardial T1 values in group 1 were 1127 ± 38 ms at pre-contrast, 308 ± 33 ms at 2 min, 392 ± 40 ms at 5 min, 463 ± 34 ms at 10 min, 500 ± 35 ms at 15 min, 525 ± 35 ms at 20 min, 553 ± 38 ms at 25 min, and 575 ± 27 ms at 30 min. Those values in group 2 were 1134 ± 43 ms at pre-contrast, 340 ± 38 ms at 2 min, 421 ± 45 ms at 5 min, 481 ± 52 ms at 10 min, 513 ± 53 ms at 15 min, 546 ± 55 ms at 20 min, 569 ± 56 ms at 25 min, and 591 ± 55 ms at 30 min. There was no correlation between myocardial T1 relaxation times and patient demographics.

## Conclusions

Post-contrast myocardial T1 mapping demonstrated lower T1 values in the group that received the single injection of contrast agent compared to the group that received divided injections with 60 sec interval to allow perfusion protocols. Pre-contrast T1 values were higher at 3 Tesla than at 1.5 Tesla regarding previously reported data.

## Funding

No financial support.

**Table 1:** 

		Group 1	Group 2
T1 values	Precontrast	1127 ± 38	1134 ± 43
	Postcontrast 2 min	308 ± 33	340 ± 38
	Postcontrast 5 min	392 ± 40	421 ± 45
	Postcontrast 10 min	463 ± 34	481 ± 52
	Postcontrast 15 min	500 ± 35	513 ± 53
	Postcontrast 20 min	525 ± 35	546 ± 55
	Postcontrast 25 min	553 ± 38	569 ± 56
	Postcontrast 30 min	575 ± 27	591 ± 55

**Figure 1:**
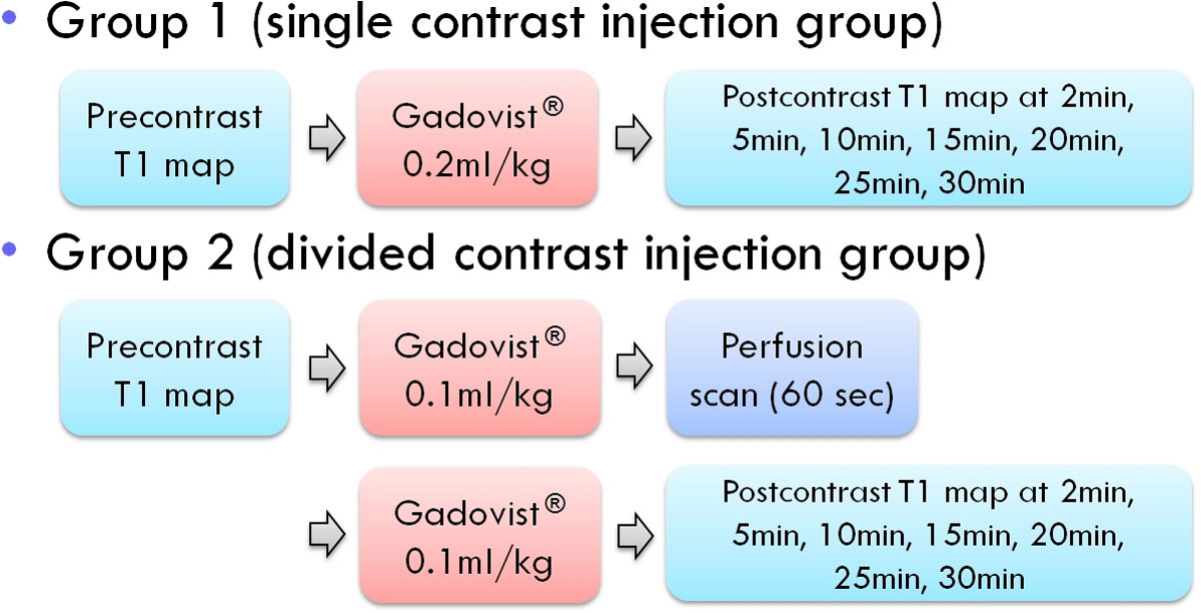
Contrast injection methods

